# Pre-Transfusion Platelet Counts and Bleeding in Very Low Birth Weight Infants

**DOI:** 10.21203/rs.3.rs-8445287/v1

**Published:** 2026-04-02

**Authors:** Ravi Patel, Elizabeth Stone, Rebecca Birch, Jeanne Hendrickson, Erika Edwards, Ruchika Goel, Eldad Hod, Matthew Karafin, Oliver Karam, Naomi Luban, Jennifer Mcintosh, Gisela Marrero-Rivera, Marianne Nellis, Jeffrey VanWormer, Nalan Yurtsever, Cassandra Josephson, Martha Sola-Visner

**Affiliations:** Emory University School of Medicine and Children’s Healthcare of Atlanta; Columbia University; Emory University; University of Vermont; Yale; Medical College of Wisconsin; Yale School of Medicine; Johns Hopkins All Children’s Hospital; Boston Children’s Hospital and Harvard Medical School

## Abstract

**Objective:**

Platelet transfusion thresholds for very low birth weight (VLBW, <1500g) infants vary widely, and the role of bleeding on platelet transfusion thresholds is unknown.

**Study Design:**

This observational birth cohort study of VLBW infants born in 7 hospitals across the US examined pre-transfusion platelet counts in infants with and without bleeding who received at least 1 platelet transfusion in the first 3 weeks of life using mixed effect linear regression models.

**Results:**

Of the 210 transfused infants, most (76%) had bleeding; intraventricular hemorrhage (IVH) was the most common (61%) bleeding type. Pre-transfusion platelet counts were not different among infants with or without bleeding diagnoses (56.3 vs 58.5×10^3^/μL, respectively; P=0.7). However, infants with severe IVH had higher platelet counts (68.9×10^3^/μL) compared to the entire cohort (53.5×10^3^/μL, P=0.01).

**Conclusion:**

Infants with or without bleeding diagnoses had similar pre-transfusion platelet counts; infants with severe IVH had higher pre-transfusion platelet counts.

## INTRODUCTION

Platelet transfusions are often administered to very low birth weight (VLBW, < 1500g) preterm infants, and there is wide variability in platelet transfusion thresholds based on surveys of practices^1–4^. In our prior analyses of Recipient Epidemiology and Donor Evaluation Study-III (REDS-III) data^3^, we found median pre-transfusion platelet counts > 50 × 10^3^/μL in extremely preterm (< 27 weeks) neonates in the first 3 postnatal weeks. The multicenter randomized trial Platelets for Neonatal Transfusions-2 (PlaNeT2), published in 2019, found that infants randomized to a higher platelet count transfusion threshold (50×10^3^/μL) had a higher incidence of death and/or major bleeding compared to infants randomized to a lower pre-transfusion platelet count (25×10^3^/μL)^5^. In addition, in our current analyses of Recipient Epidemiology and Donor Evaluation Study-IV-Pediatrics (REDS-IV-P) data, we found pre-transfusion platelet counts that were higher than expected but are limited by inclusion of neonates with bleeding that could bias pre-transfusion platelet counts^6^.

To explore the relationship between pre-transfusion platelet counts and bleeding, we compared pre-transfusion platelet counts in VLBW neonates who never had major bleeding events with those of neonates with no bleeding diagnoses. We also investigated different bleeding diagnoses and number of bleeding types. We evaluated the relationship between pre-transfusion platelet counts and the risks among these infants exposed to platelet transfusion. Furthermore, as maternal hypertensive diagnoses are associated with neonatal thrombocytopenia^7,8^, we investigated whether maternal hypertension risk factors or the presence of disseminated intravascular coagulation (DIC) leads to the use of higher or lower platelet transfusion threshold.

## MATERIALS/SUBJECTS AND METHODS

### Study Design

This was a multicenter, observational birth cohort study of VLBW infants born at hospitals that were part of the National Heart Lung and Blood Institute’s Recipient Epidemiology and Donor Evaluation Study-IV-Pediatric (REDS-IV-P) research program^9^. Included in this study were geographically distinct community hospitals and academic medical centers in the United States, including 7 hospitals with neonatal intensive care units. Participants were identified in the REDS-IV-P Vein-to-Vein (V2V) database^9^, which collects data on blood donor and transfusion recipients from all participating hospitals. Clinical data, submitted by hospital neonatology services to the Vermont-Oxford Network (VON), were also evaluated.

### Study Population

The study population included all VLBW (< 1500g) infants born at participating REDS-IV-P hospitals between April 1, 2019 and December 31, 2023; the infants were followed from birth until hospital discharge or death. Infants who received at least one platelet transfusion in the first three VON database were excluded.

### Exposures and Outcomes

The primary outcome for this study was pre-transfusion platelet counts and the primary exposure was the presence or absence of bleeding, focusing on the major sources of bleeding in VLBW infants. From a rigorously conducted prospective study on bleeding in extremely premature infants^10^, the majority of bleeding in VLBW infants includes intraventricular hemorrhage (IVH), gastric bleeding, pulmonary hemorrhage, and umbilical cord bleeding. For our study, ICD-10 codes for the birth encounter were used to identify such bleeding (**Supplemental Table 1**). Bleeding diagnoses were captured irrespective of timing relative to transfusion. We conducted a sensitivity analysis by using large volume RBC transfusion in a single day (> 20 ml/kg/day) as a surrogate measure for bleeding. In addition, we leveraged VON data on IVH not just to assess severity of IVH but also as an internal quality assessment that we were able to capture bleeding with IVH by ICD-10 codes^11^. All models were adjusted for hospital type, gestational age, post-natal age at transfusion, and repeated measures.

There were three secondary exposures for this study. One secondary exposure was death or bronchopulmonary dysplasia (BPD), defined as death during the encounter or BPD by 36 weeks per VON database. Another secondary exposure was the presence or absence of maternal hypertensive conditions of pregnancy, defined as gestational hypertension, chronic hypertension, or pre-eclampsia from maternal ICD-10 codes, since these conditions are frequent causes of early-onset neonatal thrombocytopenia. The last secondary exposure was the presence or absence of hypofibrinogenemia (defined as fibrinogen less than 150 mg/dL)^12–14^ as recorded in the infant’s chart, indicative of potential disseminated intravascular coagulopathy (DIC) around the time of platelet transfusion. Patients without a fibrinogen test were assumed to have normal fibrinogen levels.

### Statistical Analyses

Statistical analyses were conducted with SAS 9.4 and R. Descriptive analyses were performed using correlation matrices, evaluations of means, standard deviations, medians, and interquartile ranges. Descriptive analyses were used to evaluate relationships between covariates, identify potential outliers, assist with the final definitions of exposures, outcomes, and in selection/definition of covariables, and evaluate any missingness in the dataset. The primary exposure and outcome were modeled with mixed effect linear regression with adjustment for center and accounting for within-infant correlation. Covariables gestational age and day of life of transfusion were centered on zero. Interaction terms were used to evaluate if there was a statistical interaction between pre-transfusion platelet counts among infants with and without bleeding based on gestational age or postnatal age. For the secondary analysis involving death or BPD, logistic regression was used with an adjustment for VON baseline risk score (VON-RA)^15^ and center as a fixed effect to evaluate the association between mean pre-transfusion platelet count and the risks of death or BPD. VON-RA included whether the infant was small for gestational age (SGA), part of a single gestation or multiple gestation, delivery type, presence or absence of congenital anomaly, gestational age, gestational age squared, APGAR score at 1 minute, and hospital. For the secondary analysis involving maternal hypertension, mixed effect linear regression with adjustment for center and accounting for within-infant correlation was used to test whether platelet counts differed depending on the presence or absence of maternal diagnoses of hypertensive conditions of pregnancy. To model the secondary analysis involving hypofibrinogenemia, mixed effect linear regression was used to compare the mean pre-transfusion platelet count in the first three weeks of life, overall and stratified by week of age, for infants with and without hypofibrinogenemia within 48 hours of platelet transfusion. Infants without testing for fibrinogen were assumed to have normal levels of fibrinogen.

#### Ethical considerations (IRB approval, consent, etc.)

Each participating hospital obtained Internal Review Board approval for participation in the REDS-IV-P overall contract. Signed agreements were obtained from each participating hospital to allow their submitted VON data to be shared with the REDS-IV-P data coordinating center. The single central Internal Review Board for REDS-IV-P determined this study met 45 CFR 46.104 (d) category (4iii) for exempt review and granted a waiver of informed consent.

## RESULTS

We evaluated 279 VLBW infants who received a total of 686 platelet transfusions in the first 3 weeks of life (Table 1). Among these, 58 infants were excluded due to surgery and 11 were excluded due to a missing pre-transfusion platelet count within 24 hours of transfusion. Of the 210 studied infants, 39% were female. Most of these infants (63%) were born at a gestational age of less than 24 weeks, 20% were born at 24–28 weeks, 14% were born at 28–32 weeks, and 3% of the cohort was born at greater than 32 weeks of gestation. The median birth weight was 705 g (interquartile range [IQR] 550–880g), and 35% were small for gestational age (SGA). Of these infants, 76% had a bleeding diagnosis based on ICD-10 codes and VON data, with IVH occurring most commonly (61%), followed by other bleeding (26%), pulmonary hemorrhage (19%), and GI bleeding (10%). Forty-four percent of infants had only one bleeding type, 23% had two bleeding types, and 9% had three or more bleeding types; 24% had no bleeding codes documented. With respect to outcomes, 42% percent of transfused infants in this cohort died, 36% had bronchopulmonary dysplasia (BPD), and 74% died and/or had BPD. Of the 210 included infants, 111 (53%) were born to mothers who had any diagnosis for maternal hypertension, and 38 (18%) had hypofibrinogenemia within 48 hours of platelet transfusion.

The 210 infants with at least one measured platelet count within 24 hours of transfusion received a total of 649 platelet transfusions during our study period (**Supplemental Table 1**). Females received 267 platelet transfusions (41%), and most platelet transfusions (415, 64%) were given to infants born at less than 24 weeks of gestation. The majority of platelet transfusions (458, 71%) were given during the first week of life. There were 515 platelet transfusions given to the 159 infants with any bleeding diagnosis, and there were 134 platelet transfusions given to the 51 infants without bleeding. The 111 infants born to mothers with a diagnosis of maternal hypertension received 332 platelet transfusions, while the 38 infants with hypofibrinogenemia received a total 68 platelet transfusions.

### Severe intraventricular hemorrhage was associated with higher pre-transfusion platelet counts

The mean pre-transfusion platelet count for infants with platelet count testing within 24 hours of transfusion for the entire cohort was 56.6×10^3^/μL (95% CI [53.7, 59.5], Table 2, **Supplemental Table 2**). Overall, there was no significant adjusted mean difference in pre-transfusion platelet counts among infants with any bleeding when compared to infants without bleeding (estimated difference 2.2×10^3^/μL less for bleeding infants, P = 0.71). Increasing gestational age (P = 0.002) and postnatal age (P = 0.03) were associated with lower pre-transfusion platelet counts. When large volume red blood cell (RBC) transfusion (defined as 1 RBC dose of > 20 mL/kg) was used as a surrogate for bleeding, similar findings were seen for pre-transfusion platelet counts (adjusted estimated difference 5.5×10^3^/μL less for infants with large volume RBC transfusion compared to infants without, P = 0.3). When the infants’ first platelet transfusions only were assessed, there was also no significant difference between pre-transfusion platelet counts for infants with any bleeding and infants without bleeding (adjusted estimated difference 2.6×10^3^/μL less for bleeding infants, P = 0.69). To account for the fact that our analysis only included transfused infants, we also examined platelet counts in the entire cohort of 981 infants from the REDSIV-P Transfusions in Preterm Infants (TIPI) study^16^, including infants who were not transfused, and found that the 1st percentile of platelet counts for non-transfused infants was approximately 50×10^3^/μL (**Supplemental Table 3**).

**Figure 1:**
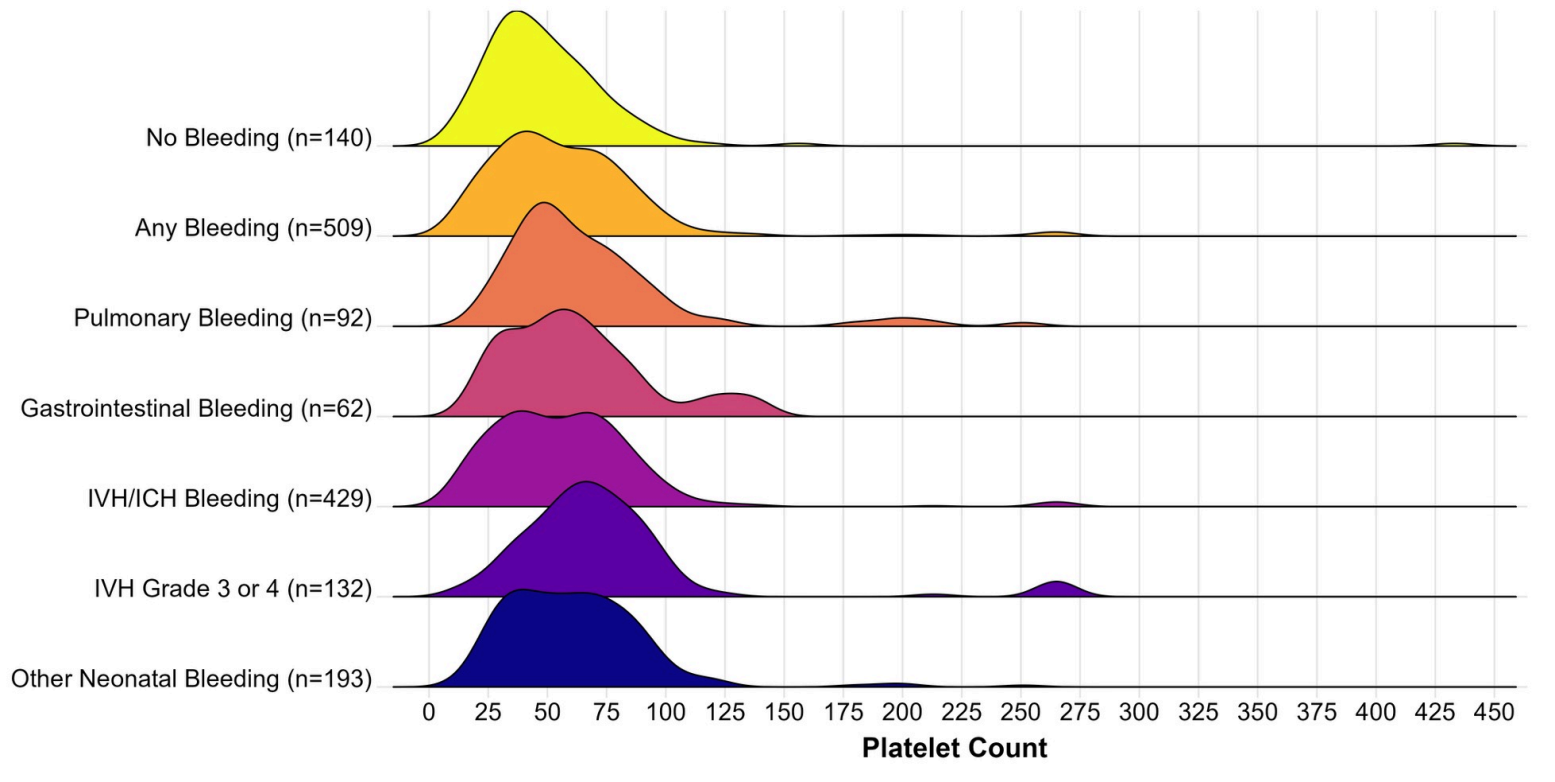
Density plot demonstrating pre-transfusion platelet count (x10^3^/μL) for infants with different bleeding diagnoses; n represents the number of platelet transfusions per category.

**Figure 2:**
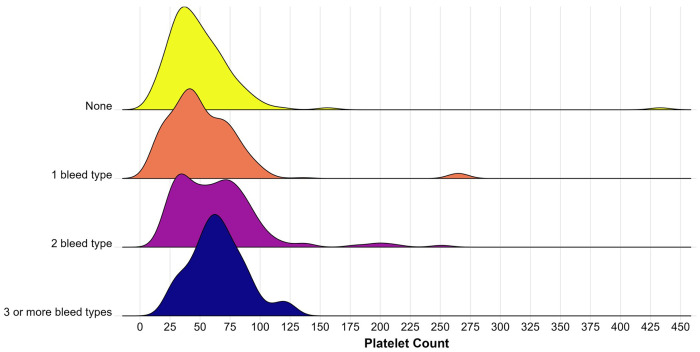
Density plot demonstrating pre-transfusion platelet count (x10^3^/μL) for infants with different numbers of bleeding diagnoses.

Among different types of bleeding identified, most bleeding types were not associated with significant adjusted mean differences in pre-transfusion platelet counts. For instance, infants with pulmonary hemorrhage had pre-transfusion platelet counts 5.5×10^3^/μL higher than infants without pulmonary hemorrhage (P = 0.40); infants with GI bleeding had pre-transfusion platelet counts 3.1×10^3^/μL higher than infants without GI bleeding (P = 0.70); infants with intraventricular hemorrhage (IVH) or intracerebral hemorrhage (ICH) had platelet counts 1.7×10^3^/μL lower than infants without IVH/ICH (P = 0.74); and infants with “other neonatal bleeding” had pre-transfusion platelet counts 0.5×10^3^/μL lower than infants without “other” bleeding (P = 0.92). Of note, only infants with severe IVH (Grade 3 or 4 by VON)^17^ had significantly higher pre-transfusion platelet counts compared to the entire cohort (adjusted estimated difference 15.4×10^3^/μL higher, P = 0.01, Fig. 1). Infants with different numbers of bleeding diagnoses did not have different pre-transfusion platelet counts compared to infants without bleeding (P values ranged from 0.57 to 0.91, Fig. 2).

### Higher pre-transfusion platelet counts were not associated with death or bronchopulmonary dysplasia

As the PLANET-2 trial demonstrated increased risk of bronchopulmonary dysplasia (BPD) in infants who received platelet transfusions with higher pre-transfusion platelet counts, we investigated whether higher pre-transfusion platelet counts were associated with worse outcomes (including death or BPD) in our study cohort. Pre-transfusion platelet counts were not associated with death (adjusted odds ratio [aOR] 0.999 [95% CI 0.991, 1.006]), BPD (aOR 1.004 [0.996, 1.012], or death *or* BPD (aOR 1.006 [0.994, 1.019]) in our patient cohort.

#### Chronic maternal hypertension, but not other maternal diagnoses of hypertension, was associated with increased pre-transfusion platelet counts

As a maternal diagnosis of hypertension has been associated with thrombocytopenia and increased platelet transfusions^7,8^, in addition to other adverse outcomes in neonates, we tested whether maternal diagnoses of hypertension were associated with higher neonatal pre-transfusion platelet counts. Pre-transfusion platelet counts did not vary for infants born to mothers with or without any maternal diagnosis of hypertension (P = 0.54), maternal diagnoses of gestational hypertension (P = 0.52), or preeclampsia or eclampsia (P = 0.64). However, infants born to mothers with diagnosis of chronic hypertension had significantly higher pre-transfusion platelet counts compared to infants born to mothers without chronic hypertension (estimated difference 17.29×10^3^/μL higher, P = 0.04).

### Hypofibrinogenemia is not associated with increased pre-transfusion platelet counts

Disseminated intravascular coagulation (DIC), caused by asphyxia at birth, sepsis, or necrotizing enterocolitis among other diagnoses, leads to platelet consumption and hypofibrinogenemia. We hypothesized that infants with DIC would have higher pre-transfusion platelet counts than infants without DIC. Using hypofibrinogenemia (fibrinogen < 150 mg/dL) as a surrogate for DIC diagnosis, and assuming that the lack of a fibrinogen test for an individual infant suggested a low clinical suspicion for DIC, we found that infants with hypofibrinogenemia did not have significantly different pre-transfusion platelet counts compared to infants with fibrinogen > 150 mg/dL and infants without fibrinogen testing (p = 0.14). When excluding infants without fibrinogen testing from the analysis, the pre-transfusion platelet count results remained the same (P = 0.37).

## DISCUSSION

In this multicenter, observational birth cohort study from 2019 through 2023 evaluating pre-transfusion platelet counts in VLBW infants, we found that presence of bleeding and different bleeding diagnoses were not associated with differences in pre-transfusion platelet counts, except for severe IVH. It is important to note that in children, other pathophysiologic risks besides platelet count likely contribute to bleeding risk, as was seen in age group analyses of the prophylactic platelet dose (PLADO) trial^18^, and these risks may extend to neonatal populations. The majority of patients (76%) who received platelet transfusions had a bleeding diagnosis at some point during hospitalization. Among the 24% of infants who did not have a bleeding diagnosis, the pre-transfusion platelet count for infants without bleeding was 58.5×10^3^/μL. This value is consistent with a snapshot of recent platelet transfusion practices across the broader cohort^16^, with or without bleeding. This is much higher than the recent AABB/ICTMG recommended platelet transfusion guidelines of 25×10^3^/μL for prophylaxis for non-bleeding neonates^5^; additional systematic reviews of the evidence are ongoing^6^.

Despite evidence that implementing more restrictive platelet transfusion guidelines in neonates does not lead to worse outcomes^19,20^, our data demonstrated higher pre-transfusion thresholds in the first two weeks of life in non-bleeding infants with thresholds outside of guideline recommendations. Barriers to implementing these new guidelines are likely multifactorial, and a recent survey suggested that providers will go against transfusion guidelines due to: (1) the assumption that the infant will reach the threshold to transfuse soon; (2) confusion around interpretation of the guidelines; and (3) the need to consult with other experts, but would otherwise err on the side of transfusion^21^. Additionally, although the PlaNeT-2 trial demonstrated better outcomes in infants with more restrictive platelet thresholds compared to liberal platelet thresholds, the trial does not address how to transfuse infants in the first week of life in thrombocytopenic neonates, as median age at randomization was 7.0 days postnatal age for the restrictive transfusion threshold, and 39% of infants in the study received at least 1 platelet transfusion before randomization^5^. The Neonatal Platelet Transfusion Threshold Trial (NeoPlaTT, NCT06676904), a US-based trial in the recruitment phase, will examine platelet transfusion thresholds in extremely low gestational age (< 27 weeks) infants in the first week of life.

Although other studies have demonstrated increased risk for death or BPD following platelet transfusion^5,22,23^, in our cohort, pre-transfusion platelet counts were not associated with the risks of death, BPD, or death *or* BPD, which may be due to the lack of difference in pre-transfusion platelet counts in the cohort except for severe IVH and maternal chronic hypertension. Of note, while our analysis was conditional on platelet transfusion, when we examined the infants who did not receive platelet transfusions, we found that the first percentile of platelet counts was approximately 50×10^3^/μL for those infants, suggesting that almost no infants would have had a threshold of less than 50×10^3^/μL for platelet transfusion. Hypertensive conditions during pregnancy are associated with placental insufficiency and thrombocytopenia^7,8^. Interestingly, infants born to mothers with gestational diagnoses of hypertension did not demonstrate differences in pre-transfusion platelet counts; however, infants born to mothers with chronic hypertension had significantly higher pre-transfusion platelet counts. These differences may be due to the different pathophysiology of chronic hypertension compared to gestational hypertension or pre-eclampsia, as infants born to mothers with chronic hypertension may have been exposed to longer periods of placental insufficiency. In addition, we used a threshold of < 150 mg/dL fibrinogen as a surrogate for DIC. This threshold is consistent with the historical lower bounds of the reference range for healthy full-term infants^14^, and may be higher than the lower bound of the reference range of < 70 mg/dL (2.5th percentile) for extremely preterm infants^12,13^. There was no difference in pre-transfusion platelet count for infants with hypofibrinogenemia.

There are several limitations of our study that must be noted. First, these analyses are observational and descriptive of practice. As such, the temporality of a platelet transfusion to a bleeding diagnosis is not known. Further, these data cannot determine causality (e.g., did a platelet transfusion increase or decrease the risk of a particular bleeding outcome or was it ordered in response to that outcome). Additionally, the approach to ascertain bleeding is limited by ICD-10 coding in the REDS database, in combination with VON clinical data. Given known limitations in ICD-10 data (including issues with diagnostic reliability)^11^, we appreciate the value the VON data adds to the bleeding diagnoses in this study. We attempted to mitigate this issue further by using large volume RBC transfusion as a surrogate for bleeding.

## CONCLUSION

In sum, platelet transfusions for VLBW infants occur at higher-than-expected pre-transfusion platelet counts, and this cannot be explained solely by the presence of bleeding. Except for severe IVH, this multicenter study showed no difference in pre-transfusion platelet counts in infants with or without a bleeding diagnosis. In addition, there was no association of pre-transfusion platelet count with death or bronchopulmonary dysplasia.

## Supplementary Material

Supplementary Files

This is a list of supplementary files associated with this preprint. Click to download.

- SupplementalTables.docx

## Figures and Tables

**Table 1:** Characteristics of VLBW infants with at least 1 platelet transfusion during first 3 weeks of life

	N	%
**Total**	262	100%
**Surgery**		
Yes (excluded)	52	20%
No	210	80%
**Sex**		
Female	82	39%
Male	128	61%
**Race**		
Asian	14	7%
Black	67	32%
White	52	25%
Not specified/Other	77	37%
**Ethnicity**		
Hispanic	67	32%
Non-Hispanic	127	60%
Unknown	16	8%
**Gestational Age**		
<24 weeks	133	63%
24–28 weeks	41	20%
28–32 weeks	29	14%
>32 weeks	7	3%
**Birth Weight, grams**		
<500	34	16%
500 to < 750	82	39%
750 to < 1000	60	29%
1000 to < 1500	34	16%
**Small for Gestational Age**		
No	133	63%
Yes	73	35%
Unknown	4	2%
**Bleeding**		
Any bleeding	159	76%
Pulmonary hemorrhage	39	19%
Umbilical hemorrhage	0	0%
Gastrointestinal bleeding	21	10%
IVH/ICH	128	61%
VON IVH (Grade ≥ 3)	42	20%
Other neonatal hemorrhage	55	26%
**Multi-bleeding**		
No bleeding	51	24%
1 bleeding type	93	44%
2 bleeding types	48	23%
3+	18	9%
**Outcomes**		
Death	89	42%
BPD	75	36%
Death or BPD	156	74%
**Maternal Hypertension**		
Any maternal hypertension	111	53%
Maternal chronic hypertension	20	10%
Maternal gestational hypertension	106	50%
Maternal preeclampsia/eclampsia	78	37%
**Hypofibrinogenemia** *	38	18%
	Mean (SD)	Median (IQR)
**Gestational Age, weeks**	26.5 (3.0)	25.9 (24.3–28.1)
**Birth weight, grams**	747 (261)	705 (550–880)

* at least one fibrinogen measure < 150 mg/dL

plt = platelet

IQR = interquartile range

**Table 2:** Pre-transfusion platelet count (x10^3/L) distribution within 24 hours of platelet transfusion, by week of life of transfusion

μ						
	Week of life	N of plt tx	Mean Plt count	LCLM	UCLM	N of Infants
**Total (excluding surgery)**	ALL (1–3)	649	56.6	53.7	59.5	210
	1	458	60.0	56.6	63.3	178
	2	125	56.1	48.3	63.8	53
	3	66	34.2	29.8	38.5	18
**No bleeding**	ALL (1–3)	134	51.1	44.2	58.0	51
	1	88	52.0	47.0	57.0	43
	2	28	61.3	31.8	90.7	12
	3	18	31.2	25.8	36.6	4
**Any bleeding**	ALL (1–3)	515	58.0	54.9	61.2	159
	1	370	61.9	57.9	65.8	135
	2	97	54.6	48.7	60.4	41
	3	48	35.3	29.6	40.9	14
**Pulmonary hemorrhage**	ALL (1–3)	92	67.8	59.4	76.2	39
	1	79	69.8	60.5	79.1	35
	2	9	62.7	34.0	91.3	4
	3	4	38.8	22.6	54.9	2
**Gastrointestinal bleeding**	ALL (1–3)	62	62.3	54.7	69.9	21
	1	37	61.2	52.4	69.9	16
	2	25	64.0	49.3	78.6	8
	3					
**IVH/ICH**	ALL (1–3)	435	57.9	54.5	61.3	128
	1	311	61.8	57.5	66.0	108
	2	87	54.0	48.0	59.9	35
	3	37	34.6	27.9	41.4	11
**Other neonatal hemorrhage**	ALL (1–3)		61.8	57.3	66.4	55
	1	150	65.8	60.4	71.2	49
	2	26	52.5	42.7	62.2	12
	3	17	41.2	32.1	50.4	7
**VON IVH (Grade ≥ 3)**	ALL (1–3)	132	75.7	67.3	84.0	42
	1	108	77.0	67.0	87.0	38
	2	21	68.6	57.1	80.2	10
	3	3	75.3	33.7	116.9	2
**Death**	ALL (1–3)	339	53.9	49.9	57.9	89
	1	221	58.4	54.0	62.8	74
	2	71	54.9	42.6	67.3	27
	3	47	30.9	25.8	36.0	10
**BPD**	ALL (1–3)	224	58.2	52.7	63.6	75
	1	164	63.9	56.9	70.8	64
	2	35	48.5	38.4	58.5	16
	3	25	34.3	29.4	39.2	6
**Death or BPD**	ALL (1–3)	513	57.2	53.7	60.7	156
	1	358	61.9	57.9	66.0	132
	2	95	54.8	45.1	64.5	41
	3	60	32.6	28.2	36.9	14

Plt Tx, platelet transfusion; LCLM, lower confidence limit of the mean; UCLM, upper confidence limit of the mean; VON IVH, Vermont Oxford Network intraventricular hemorrhage; BPD, bronchopulmonary dysplasia

## Abbreviations

VLBW: very low birth weight
REDS-III: Recipient Epidemiology and Donor Evaluation Study-III
REDS-IV-P: Recipient Epidemiology and Donor Evaluation Study-IV-Pediatric
PlaNeT-2: Platelets for Neonatal Transfusion-2
DIC: disseminated intravascular coagulation
BPD: bronchopulmonary dysplasia
VON: Vermont Oxford Network
IVH: intraventricular hemorrhage
ICH: intracerebral hemorrhage
IQR: interquartile range
aOR: adjusted odds ratio
